# Preschool Behavioral Problems: Links with Maternal Oxytocin and Caregiving Sensitivity in the Postnatal Period, and Concurrent Maternal Psychopathology and Attachment State-of-Mind

**DOI:** 10.1007/s10578-023-01529-6

**Published:** 2023-04-06

**Authors:** Jane Kohlhoff, Lisa Karlov, Mark Dadds, Bryanne Barnett, Derrick Silove, Antonio Mendoza Diaz, Valsamma Eapen

**Affiliations:** 1 Discipline of Psychiatry and Mental Health, University of New South Wales, Sydney, Australia; 2 Research Department, Karitane, Sydney, Australia; 3 Ingham Institute for Medical Research, Sydney, Australia; 4 Academic Unit of Child Psychiatry, South Western Sydney Local Health District, Sydney, Australia; 5 School of Psychology, University of Sydney, Sydney, Australia; 6 Sydney, Australia

**Keywords:** Oxytocin, Adult attachment, Sensitivity, Pre-school behavior

## Abstract

This study investigated maternal oxytocin, caregiving sensitivity and mother-to-infant bonding at 3-months postpartum as predictors of child behavior and psychological outcomes in the preschool years, when controlling for concurrent maternal negative emotional symptoms and adult attachment state-of-mind. Forty-five mother–child dyads were assessed at 3-months and 3.5 years postpartum using mix of questionnaires, observational, interview and biological methods. Results showed that lower levels of maternal baseline oxytocin at 3-months postpartum significantly predicted emotional reactivity in the child at 3.5 years. When maternal adult attachment state-of-mind and negative emotional symptoms were included, lower levels of maternal baseline oxytocin at 3-months postpartum significantly predicted withdrawn child behavior. In addition, unresolved adult attachment and maternal negative emotional symptoms were significantly associated child behavioral disturbance in a range of areas. Findings highlight maternal postnatal oxytocin as a potential indicator of children who may be more likely to show emotional reactivity and withdrawn behavior in the preschool years.

**Supplementary Information:**

The online version contains supplementary material available at 10.1007/s10578-023-01529-6.

## Introduction

Behavioral and emotional issues in the preschool years (e.g., emotional reactivity, anxiety/depression, somatic complaints, withdrawal, sleep problems, attention problems, and aggressive behaviour) [1] often represent the start of a trajectory towards mental health, social and/or academic difficulties across adolescence and adulthood [2–8]. The influence of various parenting characteristics and behaviours (e.g., lax, permissive, controlling, punitive, harsh parenting) in the development and maintenance of behavioral and emotional issues in pre-school aged children has been emphasised [9–12]. However, in seeking to better understand early developmental precursors, researchers in recent decades have also sought to investigate the role of parenting quality in the early postpartum period.

According to attachment theory, the relationship between the infant and primary caregiver in the very earliest weeks and months of life plays an important role in the child’s social, emotional and behavioral development [13, 14]. Specifically, it is hypothesised that caregiving characterised by sensitivity and appropriate responsiveness to the infant’s emotional needs shapes the infant’s social and emotional capacities, with flow-on effects to subsequent emotional and behavioural regulation [13, 14]. The interplay between early infant behaviour and caregiver sensitivity can be complex—with difficult infant temperament/behaviour often impacting a parent’s sense of bonding and connection to the child, and capacity to provide sensitive caregiving [15, 16]. Overall, however, meta-analytic studies confirm a positive association between caregiver sensitivity in the postpartum period and secure parent–infant attachment relationships (a moderate effect size, *d* = 0.24) [17], and suggest that infant attachment insecurity predicts poorer psychological functioning in the pre-school years and beyond (moderate effect sizes, *ds* = 0.37 and 0.31 for internalising and externalising disorder, respectively) [18, 19]. Relatedly, evidence also indicates that parents who have feelings of ‘closeness’ or ‘bonding’ with their infant are less likely to have children with insecure attachments and behavioral issues in the early school years [20, 21].

In recent years, there has also been an effort to identify neurobiological mechanisms underlying early mother–infant bonding and parenting sensitivity, and the oxytocin system has become a particular focus of investigation [22]. Oxytocin is a neuropeptide hormone synthesized largely in nuclei of the hypothalamus, with receptors located throughout the brain [23]. A growing body of evidence indicates the involvement of oxytocin in the initiation and maintenance of maternal bonding and caregiving sensitivity in the postnatal period [24–26]. Studies (including our own cross-sectional investigations in the sample to be examined in the current study) have shown higher levels of maternal oxytocin to be associated with stronger maternal reports of bonding with the infant [27, 28], more sensitive caregiving in the postnatal period [29, 30] and a response pattern of increased oxytocin following mother–infant interaction [29, 31, 32].

Despite evidence linking maternal oxytocin with the initiation and maintenance of maternal bonding and caregiving sensitivity in the postnatal period, current understanding of the impact of these variables on child outcomes is limited. To our knowledge, only one study, conducted by Wai et al., has examined links between maternal oxytocin and behavioral outcomes in pre-school aged children [33]. Wai et al. found that an increase in maternal oxytocin following a parent–child interaction session (child age range 2–5 years; mean 35 months) was not associated with child mental health problems. However, this study used a cross-sectional design and so conclusions regarding longitudinal pathways from early maternal oxytocin to later child behaviors cannot be made. In the only other investigation in this area, we recently showed that in a sample of 88 mother–child dyads assessed at 3-months and 12-months postpartum, maternal caregiving sensitivity at 3-months postpartum predicted infant attachment security at 12-months postpartum, but only for mothers who showed an increase in peripheral oxytocin following the parent–infant interaction [34]. To date, no studies have examined longitudinal links between maternal oxytocin in the early postpartum period and longer-term psychological outcomes for children (i.e., past the age of 12-months). Given the known prevalence and impacts of behavioral issues in pre-school aged children [2–8, 35], and the potential that the early postnatal period may provide for early intervention and prevention, this is a topic that warrants investigation.

In any investigation of longitudinal links between early parenting and later child outcomes, it is also important to consider the role of concurrent parenting environment factors. One of the parental characteristics/factors that has been linked most strongly with early childhood behavioral disturbance is parental mental ill-health. Evidence from a recent Australian data linkage study (N = 70,000 parent–child dyads), for example, showed that parental mental illness (across all diagnostic categories), was significantly associated with child externalizing and internalizing symptoms at 5 years [36]. Systematic reviews and meta-analytic studies have similarly shown strong associations between maternal emotional distress and mental health conditions in early childhood including depression and anxiety and behavioral/emotional issues [37–39]. Parenting quality is known to be the key mechanism through which parental mental ill-health impacts child psychological outcomes. Studies have shown, for example, that parental mental illness (e.g., depression and post-traumatic stress disorder) can have a detrimental impact on a parents’ capacity to provide sensitive and responsive caregiving, with flow on effects to poorer parent–child attachment relationships and compromised child social-emotional and psychological outcomes [40–44].

Another factor that has been shown to be associated with poorer social-emotional outcomes for children across infancy and into early childhood is the parent’s state-of-mind regarding their own early attachment experiences [45–48]. Attachment theory proposes that an individual’s experiences with key attachment figures, particularly in early childhood, become internalised into mental models that shape behavior in subsequent interactions and relationships, including parenting [49]. This concept was operationalized through the development and application of the Adult Attachment Interview (AAI), an hour-long interview protocol that is now considered to be the gold standard measure of adult attachment state-of-mind [50]. In the AAI, interviewees respond to a series of standardised questions asking for general descriptors of early attachment figures, along with concrete episodic examples as illustrations. AAI transcripts that are linguistically coherent are classified as secure, and those that are incoherent (e.g., poor narrative order) are classified as insecure or unresolved. The validity and reliability of the AAI has been demonstrated [51], and numerous empirical studies have shown insecure and unresolved attachment states-of-mind to be associated with poorer adverse behavioral outcomes in offspring during early childhood [45–47].

The primary aim of the current study was to examine postnatal maternal sensitivity, maternal bonding, and postnatal maternal oxytocin as predictors of behavioral issues in pre-school aged children. Given the known impacts of maternal adult attachment state-of-mind and psychopathology on parenting and child outcomes, we were also interested in examining the impact of these variables assessed concurrently with child behavior at 3.5 years. The major study hypothesis was that child behavioral issues at 3.5 years would be predicted by poorer mother–infant bonding, poorer observed maternal caregiving sensitivity, and lower levels of maternal oxytocin (baseline level and response to infant interaction) at 3 months post-partum, and that these associations would remain when maternal psychopathology (operationalized as maternal negative emotional symptom severity) and maternal adult attachment state-of-mind were also included.

## Methods

### Participants

Participants were 45 mother–child dyads who had been recruited from a public hospital antenatal clinic in Sydney, Australia, in 2011–2013, as part of a longitudinal study examining oxytocin, maternal separation anxiety and child outcomes [28, 29]. In total, 141 mother–child dyads were recruited into the longitudinal study, which involved assessments at four time points during pregnancy and the early childhood period (2nd trimester of pregnancy; 3 months postpartum, 12 months postpartum; 3.5 years post-partum). A major aim of the longitudinal study was to examine associations between adult separation anxiety and parenting and child outcomes. Hence, the sample was progressively targeted to include an equal number of participants with and participants without current adult separation anxiety symptoms as indicated by the Adult Separation Anxiety Questionnaire (ASA-27) [52]. In the final 5 months of the recruitment period (Jan 2013–May 2013), the target number of participants without separation anxiety had been reached and so participants were screened with the ASA-27 and recruited only if they scored over the threshold for current adult separation anxiety symptoms (> 22). Exclusionary criteria for the study included: mother’s age at the time of delivery of less than 18 years, non-English speaking, multiple pregnancy, not available for follow-up, and presence of an endocrine disease (e.g., thyroid disease).

The current study was conducted using data obtained at the 3-months postpartum and 3.5 years postpartum assessments. Of the 141 women who were recruited antenatally, 29 dropped out or were lost to follow up before the 3-month assessment leaving a total sample of 112 (79% retention from recruitment), and a further 8 dropped out or were lost to follow up between the 3- and 12-month assessments, leaving a sample of 106 participants at the 12-month assessment (75% retention from recruitment; 94% retention from the 3-month assessment). Of the 106 participants who took part in the 12-month assessment, 45 took part in the 3.5-year assessment (42% retention from the 12-month assessment; 40% retention from the 3-month assessment; 32% retention from recruitment).

The average age of mothers in the retained sample (*n* = 45) at recruitment was 29.2 years (*SD* = 5.84) and 44.4% were primigravid. A total of 93.3% percent were married or in a de-facto relationship, 64.4% were employed, and 68.9% had more education beyond the secondary school level. The sample was ethnically diverse, with 52.3% coming from a Caucasian background and the remainder coming mostly from Asian (25%), Arabic (11.4%) and Indian (9.1%) backgrounds. Compared to participants who participated in the 3-month postpartum assessment but who dropped out at a subsequent time point, participants who were retained in the final sample at 3.5 years were more likely to be married or in a de-facto relationship (*p* < 0.05) but there were no other significant differences (in terms of the demographic factors reported above) between participants who were retained in the final sample and those who dropped out.

### Procedure

The study was approved by the South Western Sydney Local Health District Human Research Ethics Committee. Mother–child dyads attended a clinically-based parenting research centre to complete the study measures at 3-months postpartum and 3.5-years postpartum; research assessments were conducted by trained research assistants. As detailed elsewhere [29, 34], as part of the 3-months postpartum assessment, participants completed the following tasks/steps (in sequential order): (1) completed questionnaires, (2) gave an initial blood sample for baseline plasma oxytocin analysis, (3) participated in the Still Face Procedure (SFP; [53]) with the infant, and (4) gave a second blood sample for post-parent–child interaction plasma oxytocin analysis. The infants were in the same room as the mothers during the blood sampling procedure, in most cases in a pram or on the floor. At the 3.5 year assessment, mothers participated in the Adult Attachment Interview (AAI) [50] (administered via video-conference) and completed questionnaires.

### Measures

#### Maternal Sensitivity

Maternal sensitivity at 3-months postpartum was rated from video recordings of the Still Face Procedure (SFP) [53]. The SFP is a structured experimental paradigm comprising three face-to-face interaction episodes between the mother and infant: (i) a baseline normal or ‘free-play’ interaction episode, (ii) the ‘still face’ episode in which the adult becomes unresponsive and maintains a neutral facial expression, and (ii) a ‘reunion’ episode in which the adult resumes normal interaction. The infant was placed in a bouncing chair on the floor and the mother was instructed to sit on the floor, about one meter from the child. The SFP was conducted in a small room fitted with cameras fixed to film the bodies and faces of both mother and infant. The researcher sat outside the room, giving two knocks on the door to signal the start/end of each still face episode. In the current study, episode 1 (‘free play’) was 3 min in length, episode 2 (‘still face’) was 90 s and episode 3 (‘reunion’) was 2 min in length. A trained coder who was blind to participants’ scores on study variables used the ‘good-poor’ scale of the Global Rating Scales of Mother–Infant Interaction (GRS) [54] to rate maternal sensitivity during the reunion episode, with higher scores indicating more optimal maternal caregiving (referred to hereafter in this paper as ‘maternal sensitivity’). In its initial development, the GRS was shown to possess good construct/predictive validity and inter-rater reliability (intra-class correlations for the maternal scales > 0.94) and subsequent studies have demonstrated its validity and reliability in other samples [55, 56]. In the current study, 10% of cases were double coded (intra-class correlation coefficient = 0.78).

#### Mother-to-Infant Bonding

Mother-to-infant bonding at 3-months postpartum was assessed using the Mother-to-Infant Bonding scale (MIBS) [57], an 8-item, self-report measure assessing the mother’s feelings towards her baby. Each of the MIBS items comprises an adjective that describes how they feel about their infant (e.g., ‘resentful’ or ‘protective’), rated on a four-point scale. A total score is calculated, with higher scores indicating worse mother-to-infant bonding. The MIBS has been shown to have good internal consistency (Cronbach’s alpha = 0.71) [57] and to correlate significantly with others measures of the same construct (e.g., the Parental Bonding Questionnaire, *r* = 0.48, *p* < 0.05) [58]. In the current sample, the internal consistency was high (Cronbach’s alpha 0.91).

#### Maternal Oxytocin

Maternal peripheral oxytocin at 3-months postpartum was assessed from blood samples, analysed at a commercial facility based in Germany using the radioimmunoassay (RIA) method. Blood samples were collected in vacutainer tubes and centrifuged at 3000 RPM for 10 min. Plasma was then pipetted and stored at − 20 °C until extraction, at which time 20 mg heat-activated (700 uC) LiChroprep Si 60 (Merck) in 1 ml distilled water was added to each sample, mixed for 30 min and centrifuged. Pellets were then washed with distilled water and 0.01 N acidic acid and mixed in 60% acetone. Evaporated extracts were kept at − 20 °C. Assay buffer (0.05 ml) was added and oxytocin assessed using a radioimmunoassay. Antibody (0.05 ml) and l-labeled tracer (0.01 ml) were added to each aliquot and after a 3-day incubation period, unbound radioactivity was precipitated by activated charcoal. All evaporated plasma extracts were treated identically. The detection limit was in the 0.5 pg/sample range and antiserum cross-reactivity was less than 0.7%.

#### Maternal Negative Emotional Symptoms

The mother’s negative emotional symptoms at 3.5 years postpartum were assessed using the Depression, Anxiety and Stress Scales (DASS) [59]. The DASS is a validated self-report scale designed to assess the severity of current depressive, anxiety and stress symptoms. In the current study the 21-item DASS was used, and scores from the three subscales were summed to form a total composite score representing negative emotional symptoms. In the current sample, the internal consistency was high (Cronbach’s alpha 0.93).

#### Maternal Adult Attachment

The mother’s adult attachment state-of-mind at 3.5 years postpartum was assessed using the Adult Attachment Interview (AAI) [60], a validated, structured interview protocol. The interview covers topics including relationships with parents in early childhood, loss and trauma, impact of early experiences on adult functioning, and current relationships with parents and children. Transcripts are coded by trained, accredited coders and can be classified using both 3-way and 4-way classification systems. In the 3-way system, transcripts are coded as ‘Secure’ (F), ‘Dismissing’ (Ds) or ‘Preoccupied’ (E) regarding past and current attachment relationships. Autonomous or ‘secure’ transcripts are characterized by a coherent, consistent and appropriately succinct account of attachment-related experiences, whereas insecure transcripts (dismissive and preoccupied) tend to contain violations of the maxims of quantity, quality, relevance and manner. Dismissive speakers attempt to deny or minimize the significance of attachment-related experiences, for example insisting on lack of memory for childhood experiences or providing idealized accounts of early relationships with parents that are not supported by evidence. In contrast, preoccupied individuals tend to show an angry, passive, fearful or confused preoccupation with early attachment experiences, reflected in the use of lengthy, grammatically entangled descriptions and jargon and nonsense words. In the four-way AAI coding system, when there is evidence of lapses of monitoring or reasoning or discourse while discussing potentially traumatic experiences, transcripts are given a primary classification of ‘Unresolved/disorganized’ (Ud) regarding previous abuse or loss experiences. For transcripts that are not Ud, the ‘organized’ classification (i.e., F, Ds or E) becomes the primary classification. In the current study, transcripts were coded by a trained and certified-reliable AAI coder, who was masked to study design, hypotheses and all other study variables. A second AAI coder, who was also certified-reliable, coded 20% of the transcripts to ensure coding reliability. The percentage agreements for the 3-way and 4-way coding systems were 77% and 88%, respectively. In cases where there were coding disagreements, differences were resolved via consensus discussion.

#### Child Behavior

Parent-reported child behavior at 3.5 years postpartum was assessed using the Child Behavior Checklist for ages 1.5–5 years (CBCL) [1], a parent/teacher report scale designed to measure behavioral functioning in children aged 1.5–5 years. The CBCL comprises 99 items, each rated in terms of the frequency with which the child displays given problem behaviors on a scale of 0–2 (higher scores indicating presence of the behavior). CBCL scores can be summed to yield seven syndrome scores: emotionally reactive, anxious/depressed, somatic complaints, withdrawn, sleep problems, attention problems, and aggressive behavior. The internal consistency and test–retest reliability of the CBCL have been demonstrated [1]. In the current sample, the Cronbach’s alpha for the somatic syndrome scale was 0.57; for all other syndrome scales alphas ranged from 0.67 to 0.89.

### Analysis

Two oxytocin variables were used: (i) baseline oxytocin level and (ii) oxytocin response, defined as the change in maternal oxytocin level from baseline following the mother–infant interaction. An oxytocin response score was calculated by subtracting the baseline oxytocin level from post-SFP oxytocin level. A positive oxytocin response score thus indicated an increase in maternal oxytocin from baseline to post-SFP (with a higher positive score indicating a larger increase) and a negative oxytocin response score indicated a decrease in maternal oxytocin from baseline to post-SFP (with a lower negative score indicating a larger decrease). Two adult attachment state-of-mind variables were used: (i) Insecure attachment state-of-mind (3-way), coded as 1 = insecure (primary classification of Ds or E) and 0 = secure (primary classification of F) and (ii) Unresolved attachment state-of-mind (4-way), coded as 1 = unresolved (primary classification of Ud) and 0 = organised (primary classification of F, Ds or E).

Analyses were conducted using JASP [61]. As a preliminary step, bivariate correlations were conducted to examine associations between the CBCL syndrome scales and the variables of interest (maternal baseline oxytocin; maternal oxytocin response; maternal sensitivity, mother–infant bonding [MIBS total], insecure attachment state-of-mind (AAI-Insecurity]; unresolved attachment state-of-mind [AAI-unresolved]; and negative maternal emotionality [DASS total]).

Seven multiple linear regressions were then conducted to test associations between the seven independent variables (IVs; maternal sensitivity, MIBS-total, maternal baseline oxytocin, maternal oxytocin response, AAI-Insecurity, AAI-unresolved, DASS total) and each of the dependent variables (DVs; CBCL syndrome scales). The regression models were all conducted in two steps: Step 1 contained variables measured at 3-months postpartum (‘historical variables’; maternal sensitivity, MIBS-total, maternal baseline oxytocin, maternal oxytocin response), and Step 2 added the three variables measured at 3.5 years postpartum, concurrently with the assessment of child behavioral issues (‘concurrent variables’; AAI-Insecurity, AAI-unresolved, DASS total).

## Results

### Bivariate Correlations

Table 1 shows descriptive statistics and results of the correlational analysis. Higher maternal baseline oxytocin at 3-months postpartum was associated with lower levels of emotional reactivity in the child at 3.5 years (*p* < 0.05). There was a correlation between insecure adult attachment and higher levels of child aggression at 3.5 years (*p* < 0.05). Unresolved adult attachment state-of-mind at 3.5 years was also associated with higher scores on five of the CBCL syndrome scales at 3.5 years: emotional reactivity, anxious/depressed, withdrawn, sleep difficulties, and aggression (all *p* < 0.05). The DASS total score at 3.5 years was positively correlated with five of the CBCL syndrome scale scores: emotionally reactive, anxious/depressed, sleep, attention, and aggression (all *p* < 0.05).

**Table 1:** Bivariate correlations and descriptive statistics

	Emotionally reactive^b^		Anxious/Depressed^b^		Withdrawn^b^		Somatic^b^		Sleep^b^		Attention^b^		Aggression^b^		*n*	*Mean*	*SD*
	*r*	*p*	*r*	*p*	*r*	*p*	*r*	*p*	*r*	*p*	*r*	*p*	*r*	*p*			
Oxytocin baseline^a^	− 0.35*	0.02	− 0.17	0.27	− 0.24	0.12	− 0.07	0.66	0.00	0.99	− 0.13	0.42	− 0.26	0.09	45	7.66	8.54
Oxytocin response^a^	0.05	0.78	− 0.04	0.79	0.06	0.68	0.03	0.86	− 0.23	0.12	0.00	0.99	0.16	0.31	45	− 0.68	3.68
Maternal sensitivity^a^	− 0.12	0.42	− 0.01	0.93	0.05	0.76	− 0.22	0.15	− 0.14	0.37	− 0.12	0.43	− 0.15	0.33	45	4.03	0.37
MIBS^a^	0.00	0.99	0.08	0.63	− 0.07	0.64	− 0.08	0.62	− 0.08	0.62	0.04	0.81	− 0.02	0.88	44	1.32	1.91
AAI-insecure^b^	0.17	0.31	0.30	0.07	0.26	0.11	0.27	0.10	0.06	0.74	0.04	0.83	0.34*	0.04	37	0.73	0.45
AAI-unresolved^b^	0.44*	0.00	0.40*	0.01	0.44*	0.01	0.29	0.09	0.35*	0.03	0.29	0.08	0.44*	0.00	37	0.43	0.50
DASS total^b^	0.43*	0.00	0.35*	0.02	0.16	0.29	0.23	0.13	0.41*	0.01	0.30*	0.05	0.42*	0.01	45	10.80	9.28
n	45		45		45		45		45		45		44				
Mean	2.09		2.29		2.29		1.84		3.09		3.80		10.39				
SD	2.34		2.18		2.53		1.88		2.60		2.71		7.36				

*MIBS* Maternal to Infant Bonding Scale; *AAI* Adult Attachment Interview; *DASS* Depression, Anxiety, Stress Scales

*p < 0.05

^a^Measured at 3-months postpartum

^b^Measured at 3.5 years postpartum

### Multiple Linear Regressions

The results of the multiple linear regression stepwise analyses are shown in Table 2. For each regression, step 1 contained variables measured at the ‘historical’ time-point (3-months postpartum) and step 2 contained the two variables of interest measured at 3.5 years postpartum (assessed concurrently with the child behavioral outcomes). In all models, the only significant finding at step 1 was that lower levels of baseline maternal oxytocin at 3-months postpartum predicted higher levels of child emotional reactivity at 3.5 years (*p* < 0.05). When all IVs were included (step 2), lower baseline levels of maternal oxytocin at 3-months postpartum predicted higher levels of withdrawn child behavior at 3.5 years (*p* < 0.05) and an unresolved adult attachment state-of-mind at 3.5 years positively predicted higher scores on four CBCL syndrome scales at the same time point: emotional reactivity (*p* < 0.01), anxious/depressed (*p* < 0.05), withdrawn (*p* < 0.05), and aggression (*p* < 0.05). In addition, higher levels of negative emotional symptoms in mothers at 3.5 years predicted independently predicted higher scores on four CBCL syndrome scales: emotional reactivity (*p* < 0.01), anxious/depressed (*p* < 0.05), sleep (*p* < 0.05), and aggression (*p* < 0.05).

**Table 2:** Multiple linear regression models predicting scores on the seven CBCL syndrome scales at 3.5 years

	Emotionally reactive^b^	Anxious/Depressed^b^	Withdrawn^b^	Somatic^b^	Sleep^b^	Attention^b^	Aggression^b^
Step 1: historical factors							
*F* (4,39)	1.511	0.523	1.081	0.592	0.796	0.249	0.813
*p*	0.218	0.719	0.379	0.670	0.535	0.909	0.525
*R*^2^	0.134	0.051	0.100	0.057	0.075	0.025	0.079
*RMSE*	2.310	2.247	2.540	1.937	2.586	2.840	7.512
Step 2: concurrent factors							
*F* (7,28)	4.731*	3.190*	2.206	1.524	1.927	1.122	3.861*
*p*	0.001	0.013	0.065	0.200	0.103	0.378	0.005
*R*^2^	0.542	0.444	0.355	0.276	0.325	0.219	0.491
*RMSE*	1.554	1.636	2.269	1.840	2.436	2.663	5.241
Step 1: historical predictor variables							
Oxytocin baseline^a^							
*b*	− 0.109*	− 0.062	0.053	0.007	0.001	− 0.033	− 0.174
*p*	0.028	0.195	0.060	0.872	0.991	0.582	0.275
Oxytocin response^a^							
*b*	− 0.042	− 0.072	0.111	0.028	− 0.161	− 0.025	0.216
*p*	0.682	0.466	0.861	0.746	0.164	0.842	0.520
Maternal sensitivity^a^							
*b*	0.239	0.483	1.158	− 1.193	− 0.824	− 0.601	− 1.292
*p*	0.822	0.640	0.272	0.185	0.489	0.645	0.710
MIBS^a^							
*b*	− 0.036	0.074	0.205	− 0.072	− 0.054	0.051	− 0.214
*p*	0.848	0.685	0.477	0.647	0.796	0.826	0.727
Step 2: concurrent predictor variables							
Oxytocin baseline^a^							
*b*	− 0.064	− 0.002	− 0.104*	0.028	0.007	− 0.037	− 0.116
*p*	0.075	0.951	0.049	0.496	0.905	0.536	0.329
Oxytocin response^a^							
*b*	− 0.041	− 0.125	0.054	− 0.061	− 0.147	0.085	0.473
*p*	0.612	0.151	0.651	0.528	0.253	0.542	0.092
Maternal sensitivity^a^							
*b*	0.220	− 0.159	1.767	− 1.763	− 0.181	0.509	0.440
*p*	0.778	0.847	0.129	0.065	0.882	0.704	0.867
MIBS^a^							
*b*	− 0.025	0.177	− 0.075	− 0.073	− 0.144	0.060	0.005
*p*	0.864	0.245	0.719	0.669	0.522	0.805	0.992
AAI-insecure^b^							
*b*	− 0.397	0.776	0.202	0.862	− 1.062	− 0.916	1.282
*p*	0.558	0.281	0.838	0.287	0.321	0.432	0.575
AAI-unresolved^b^							
*b*	1.687*	1.289*	2.038*	0.772	1.729	1.743	4.448*
*p*	0.007	0.043	0.022	0.268	0.066	0.089	0.030
DASS total^b^							
*b*	0.097*	0.074*	0.021	0.035	0.118*	0.072	0.242*
*p*	0.002	0.017	0.611	0.293	0.012	0.142	0.015

*MIBS* Maternal to Infant Bonding Scale; *AAI* Adult Attachment Interview; *DASS* Depression, Anxiety, Stress Scales

*p < 0.05

^a^Measured at 3-months postpartum

^b^Measured at 3.5 years postpartum

## Discussion

This study represents the first longitudinal examination of maternal postnatal oxytocin and caregiving (sensitivity and mother-to-infant bonding) at 3-months postpartum as early predictors of child behavior problems in the preschool years (3.5 years postpartum). While there was little evidence of an association between early maternal oxytocin and child behavioural outcomes at 3.5 years, the one exception was that of child withdrawn behaviour.

The finding that maternal oxytocin predicted withdrawn behavior in the preschool-aged child when the mother’s concurrent negative emotional symptoms and adult attachment state-of-mind were included highlights the strength of this association and indicates the unique predictive role that maternal oxytocin may have for longer-term child social and psychological outcomes. It is also of note that maternal postnatal oxytocin predicted emotionally reactive behavior in the child at 3.5 years. This effect was no longer significant when maternal negative emotional symptoms and adult attachment state-of-mind were entered into the model. While it is unknown whether different results would have been obtained had the sample size been larger, the fact that the *p* value (*p* = 0.07) was so close to significance suggests that with more participants and thus greater power, this effect may possibly have remained significant in the final model. Young children who are shy or socially withdrawn are known to be at risk of a range of negative outcomes including socio-emotional difficulties, peer-relationship difficulties, and academic challenges [62]. Similarly, children who are emotionally reactive are more likely to experience sleep difficulties, internalizing and externalizing problems, and social adjustment issues [63–66].

Results of this study are thus significant because they highlight maternal postnatal oxytocin as a potential early indicator of which children may be more likely to struggle with withdrawn behavior or emotional reactivity in the preschool years. In addition, they also have implications for the prevention of early childhood mental problems. While a number of successful interventions aimed at improving outcomes for socially withdrawn and/or emotionally reactive preschool aged children have been developed (e.g., [67]), results of this study suggest potential avenues for early interventions that may be able to *prevent* the development of shyness/withdrawn behavior in preschool aged children. Given oxytocin’s known role in the establishment of emotional interpersonal bonds, early parenting interventions that promote touch and affection among mothers with low levels of oxytocin are likely to be of significant benefit—not only to the early parent–infant relationship but also in the prevention of withdrawn behavior in the child during the preschool years.

Another contribution of this study is the finding of associations between unresolved adult attachment state-of-mind and child behavioral issues, and between maternal negative emotional symptoms and child behavioral issues, across a broad range of syndrome areas. The high *b* values obtained (particularly for child aggression) indicate strong effects. These findings align with research from the wider literature about the impact of adult attachment and maternal mental health on child behavioral outcomes [37, 39, 45–47] and the clinical implications of this are significant. Recommended interventions for pre-schoolers with externalizing and internalizing behavior issues have tended to be based in behavioral and cognitive-behavioral theories [68–70]. Results of this study are important, however, because they suggest that parent-focused programs that aim to increase parental mental health and enhance parental awareness of past and present adult attachment relationships, and to process unresolved trauma and loss, may also be of benefit. Evidence suggests that unresolved and insecurely attached adults can achieve “earned” security [71, 72] through relational approaches centring around a safe and stable therapist-parent relationship/working alliance [73], and so programs of this kind for parents of pre-schoolers with behavioral issues are likely to be of benefit.

The study had a number of strengths including use of gold standard measures and procedures including the SFP and AAI, but various limitations need to be acknowledged. The major limitation of the study is the small sample size. While small samples have been reported in similar studies given the difficulties with following parent–child dyads up over multiple time points (e.g., [45]) it must be noted that the small sample size in this study (*n* = 45) meant that the Step 2 models with seven IVs were slightly underpowered to detect significant results. It was, however, considered theoretically important to include all of the IVs at this step, and the large *R*^2^ values obtained suggest that these are highly relevant models. Results should be nevertheless be interpreted with caution and used as proof of concept for future larger studies (which should also include adjustments for multiple comparisons). An additional study limitation relates to the failure to account for other potentially relevant control variables (e.g., genetics, child temperament/difficulty, parenting styles, previous maternal postpartum depression). Given the small sample size in this study, it was appropriate to include a large number of predictor variables. This means, however, that results must be interpreted accordingly, and further replication studies with larger samples and a greater range of predictor variables, are warranted. In particular, the complex transactional nature of early parent and child behaviours and biology [74, 75] makes it difficult to disentangle the role of different contributing factors. For example, challenging infant behaviours in the early postpartum period (e.g., levels of crying, sleeping and fussing) may influence maternal oxytocin, which may in turn influence parenting sensitivity and child behavioral outcomes. Conversely, lower levels of maternal oxytocin and sensitivity may contribute to early difficult infant behaviour, with flow on effects to later child behavioral outcomes. Future studies should seek to account for early infant temperament, as a way of better understanding the influence of this important potentially relevant factor. A final limitation was the use of RIA for oxytocin analysis as this is now slightly out-dated, with most recent studies now using the ELISA method. At the time of data collection, RIA was a methodology that was being used by other researchers in the same field [31].

## Summary

Taken together, results of this longitudinal study suggest that mothers with lower postnatal oxytocin levels are more likely to have children who display withdrawn behavior in the preschool years. In addition, in alignment with previous research, mothers with an unresolved adult attachment state-of-mind and higher levels of negative emotional symptoms are more likely to have children who develop behavioral issues in a range of domains in the preschool years. These results have two potential clinical implications: First, that there may be scope in the future for identifying women with lower levels of oxytocin in the postnatal period and providing them with parenting support across the early years of the child’s development, with possible positive benefits for the child in the preschool years. To pave the way for this type of work, future research will be required to confirm findings of this study, and to determine thresholds and optimal measurement approaches. Second, interventions focusing on helping mothers process and resolve difficult early attachment, loss or trauma experiences, and which address maternal mental health, are likely to be of significant benefit to their children, and may be a useful adjunct to parenting interventions focusing on improving preschool behavior.

## Supplementary Information

Below is the link to the electronic supplementary material.

Supplementary file1 (DOCX 22 kb)
