# Supplementary material for: Preschool Behavioral Problems: Links with Maternal Oxytocin and Caregiving Sensitivity in the Postnatal Period, and Concurrent Maternal Psychopathology and Attachment State-of-Mind
Source: Child Psychiatry Hum Dev. 2023 Apr 6;55(6):1736–46. doi: 10.1007/s10578-023-01529-6 (PMC11485215; doi:10.1007/s10578-023-01529-6)
Supplement: Supplementary file 1 — Supplementary file1 (DOCX 22 kb) [file 10578_2023_1529_MOESM1_ESM.docx]

Supplementary Figure 1. Participant flow through the study
